# Expression profile of a *Caenorhabditis elegans* model of adult neuronal ceroid lipofuscinosis reveals down regulation of ubiquitin E3 ligase components

**DOI:** 10.1038/srep14392

**Published:** 2015-09-23

**Authors:** Hannah V. McCue, Xi Chen, Jeff W. Barclay, Alan Morgan, Robert D. Burgoyne

**Affiliations:** 1 Department of Cellular and Molecular Physiology, Institute of Translational Medicine, University of Liverpool, Crown St, Liverpool L69 3BX, UK

## Abstract

Cysteine string protein (CSP) is a chaperone of the Dnaj/Hsp40 family of proteins and is essential for synaptic maintenance. Mutations in the human gene encoding CSP, DNAJC5, cause adult neuronal ceroid lipofucinosis (ANCL) which is characterised by progressive dementia, movement disorders, seizures and premature death. CSP null models in mice, flies and worms have been shown to also exhibit similar neurodegenerative phenotypes. Here we have explored the mechanisms underlying ANCL disease progression using *Caenorhaditis elegans* mutant strains of *dnj-14*, the worm orthologue of DNAJC5. Transcriptional profiling of these mutants compared to control strains revealed a broad down-regulation of ubiquitin proteasome system (UPS)-related genes, in particular, components of multimeric RING E3 ubiquitin ligases including F-Box, SKR and BTB proteins. These data were supported by the observation that *dnj-14* mutant worm strains expressing a GFP-tagged ubiquitin fusion degradation substrate exhibited decreased ubiquitylated protein degradation. The results indicate that disruption of an essential synaptic chaperone leads to changes in expression levels of UPS-related proteins which has a knock-on effect on overall protein degradation in *C. elegans.* The specific over-representation of E3 ubiquitin ligase components revealed in our study, suggests that proteins and complexes upstream of the proteasome itself may be beneficial therapeutic targets.

Protein misfolding and aggregation have been implicated in the progression of a number of neurodegenerative disorders including Alzheimer’s disease, Huntington’s disease, Parkinson’s disease, and prion disorders^1^. It is therefore thought that maintaining correct protein homeostasis is important in the prevention of neurodegeneration. Various studies have identified genetic and pharmacological interventions which confer a neuroprotective effect by targeting molecular chaperones, proteasomes and autophagy pathways^1^,^2^,^3^.

Cysteine String Protein (CSP)^4^ is a co-chaperone of the DnaJ/Hsp40 family of proteins present on synaptic and secretory vesicles which is encoded by the DNAJC5 gene in humans^5^,^6^,^7^,^8^. CSP is essential for synaptic maintenance and mutations in the DNAJC5 gene cause adult neuronal ceroid lipofuscinosis (ANCL)^9^,^10^,^11^,^12^. Clinical symptoms of ANCL include generalized epilepsy, movement disorders, progressive dementia and premature death^11^.

Mouse *dnajc5* null mutants are also characterized by age-dependent sensorimotor dysfunction, neurodegeneration and premature mortality^13^. In particular, studies using CSP-deficient mice have revealed an activity-dependent impairment of synaptic function suggesting that CSP may act to maintain functional SNARE complexes which are essential for neurotransmitter release^14^. The fastest synaptic degeneration occurs in photoreceptor ribbon synapses^15^ and GABAergic synapses^16^ whereas degeneration at neuromuscular junctions develops more slowly^17^. Indeed, there is a 50% decrease in levels of the essential SNARE protein, SNAP-25, in CSP knockout (KO) mice and a corresponding decrease in SNARE complex assembly^18^. In addition, overexpression of SNAP-25 in CSP KO mice was able to rescue the neurodegenerative phenotype^19^. These findings have suggested that SNAP-25 is the major client for the chaperone activity of CSP. It is likely that SNAP-25 adopts abnormal conformations during synaptic activity, which, if not corrected by CSP chaperone activity, impair SNARE complex assembly leading to neurodegeneration^20^. In the absence of CSP these abnormal conformers are shown to be ubiquitylated and targeted for proteasome degradation^21^. In addition to its function in synaptic vesicle exocytosis, a role in endocytosis and synaptic vesicle recycling has also been suggested and CSP has been shown to regulate the polymerization of dynamin I^17^,^22^.

CSP-deficient models have also been developed in flies and worms^23^,^24^,^25^,^26^. These animals exhibit progressive neurodegeneration and reduced lifespan indicating a conserved function for CSP from invertebrates to higher mammals. *Caenorhabditis elegans* models have proven useful for the study of neurodegenerative diseases due to their genetic tractability, cost effectiveness and short life cycle^27^,^28^,^29^. Often, however, in the absence of a worm orthologue, human genes containing disease-associated mutations are exogenously, overexpressed in these models^30^,^31^. Models of CSP dysfunction in worms, on the other hand, are based on mutations in an endogenously encoded CSP orthologue, *dnj-14*, which exhibits over 40% identity within the J-domain and cysteine string domain when compared to human CSP^25^. *C. elegans* models of CSP dysfunction more closely mimic the progression of the disease state in humans due to the progressive nature of the degenerative phenotypes into adulthood, whereas fly and mouse null models have proven lethal at a much earlier age^23^,^13^. Additionally, *C. elegans* mutants have been maintained over hundreds of generations which may allow selective pressures to exert effects and reveal compensatory changes in gene expression that might not be observed in mouse KO models that have been maintained for a single generation only.

In CSP-deficient mice proteasome inhibitors were found to prevent the decrease in SNAP-25 levels and SNARE complex assembly and to increase life-span^21^. As CSP function is conserved from worms to man, elucidation of the mechanisms and regulators of CSP-dependent pathways in worms may illuminate general aspects of normal neuroprotective pathways that could be targeted in the treatment of neurodegenerative diseases. We have, therefore used the model organism, *C. elegans,* to identify potential novel regulators of mutant-CSP mediated neurodegeneration. Transcriptional profiling of two *dnj-14* mutant variants revealed a broad downregulation of ubiquitin-proteasome system (UPS) related genes. In particular, genes encoding several key components of multimeric RING finger E3 ubiquitin ligase complexes were overrepresented in the down-regulated transcripts. In support of this we observed a decrease in ubiquitylated protein degradation in *dnj-14* mutant nematodes.

## Results

### Experimental design

The *C. elegans* genome encodes a single homologue, *dnj-14*, of the human DNAJC5 gene^25^. We have previously characterised two mutant *C. elegans* strains which contain variants of the *dnj-14* gene^25^. One variant, *dnj-14(ok237)*, contains a 2229 bp deletion that eliminates the majority of the *dnj-14* coding region, its putative promoter and part of the adjacent *glit-1* gene. The other variant, *dnj-14(tm3223)*, contains a 233 bp deletion and an insertion of five adenines within exon 2 of *dnj-14*. Previous work has revealed that both mutant strains exhibit shortened lifespan, age-dependent impairment of locomotion and neurotransmission, and neurodegeneration of sensory neurons coupled with a progressive decline in chemosensory behaviour^25^.

To gain further insight into the genetic mechanisms underlying the pathologic effects of mutations in *dnj-14* and thus better understand how wild-type *dnj-14* helps to protect against neurodegeneration in wild-type animals, a whole genome *C. elegans* microarray was performed (ArrayExpress accession number: E-MTAB-3147). Global transcription levels of the two *dnj-14* mutant strains were compared to two control strains, N2 (Bristol) wild-type and CZ1200 (juIs76 [p*unc-25*::GFP + *lin-15*(+)] II), which was selected as a superficially wildtype strain that was readily available in the lab. CZ1200 stably expresses [p*unc-25*::GFP + *lin-15*(+)]II, which rescues the effects of a mutation, n765, in the gene encoding the transcription factor *lin-15*. The use of both two control and two experimental strains ensured a high level of stringency when identifying differentially expressed genes (DEGs) in the resultant dataset. Previous work identified a progressive chemosensory defect that is already severe in day 6 adult worms but which precedes visible abnormalities in sensory neuron morphology^25^. Therefore total RNA samples from the four *C. elegans* strains were isolated from synchronized worms which had been aged until day 6 of adulthood. This allowed us to assess global transcriptional changes that occur specifically at the onset of neuronal signalling impairment but prior to neuronal cell damage as evidenced by accumulation of aggregates and loss of neurons in *dnj-14* mutants expressing GFP pan-neuronally^25^.

### Differentially regulated genes in dnj-14 mutant strains

**Figure 1:**
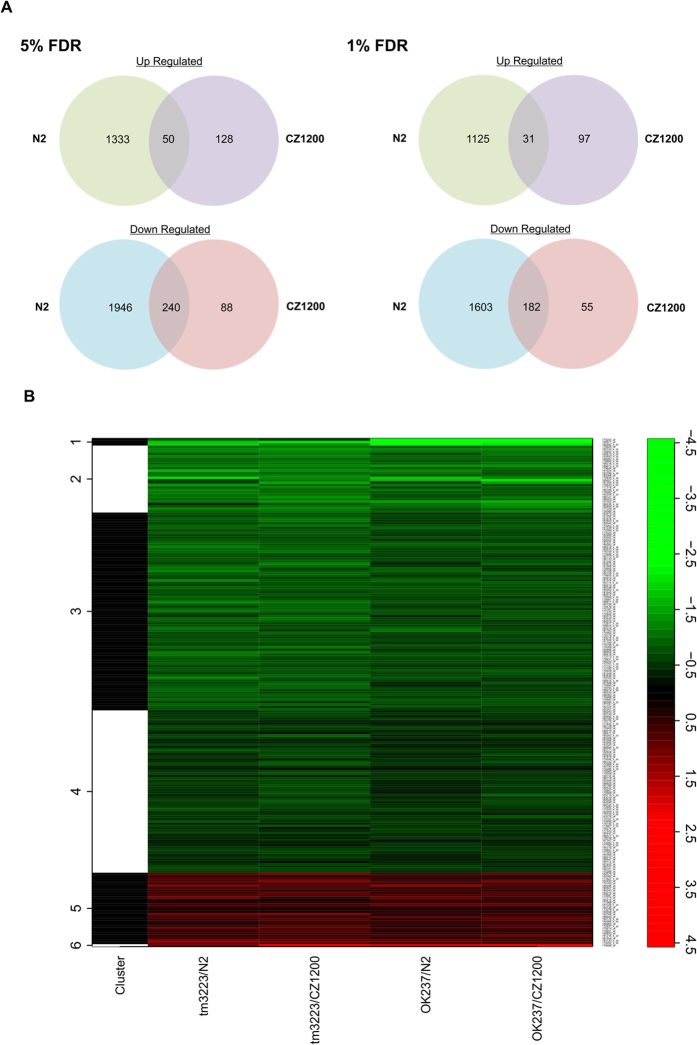
Venn Diagrams and a heatmap representing differentially regulated genes. (**A**) Venn diagrams illustrating the number of up and down-regulated genes for both the 5% and 1% FDR datasets in *dnj-14* mutants compared to either N2 or CZ1200 and the number of common genes between the two control strains. (**B**) Microarray data heatmap illustrating the log2 fold change of DEGs (FDR < 0.01) common to both, tm3223 and ok237, *dnj-14* mutant strains compared to N2 and CZ1200 control strains. Each row represents one of 213 genes, and each column represents the comparison of each mutant strain to each control strain. The column on the left clusters the samples based on similarity in fold change among the 4 samples. Green color represents a lower log2 fold change while red illustrates a higher log2 fold change. The scale on the right matches colours to log2 fold change values.

Twelve samples were hybridized to arrays consisting of 22,625 probes representing 20,149 predicted *C. elegans* open reading frames. The data were analysed in order to identify genes which were commonly up- or down-regulated in both *dnj-14* mutant strains compared to either of the two control samples (Supplementary Table S1 and Supplementary Fig. S1). Both 5% and 1% false discovery rate (FDR) datasets were analysed in order to ensure the highest confidence in the observed enriched genes and pathways whist ensuring there was no loss of representation of DEGs at higher stringency levels. At 5% FDR, 3569 DEGs were revealed compared to N2 (Bristol) and 506 DEGs compared to CZ1200. Further analysis of these data identified 50 Up-regulated and 240 down-regulated DEGs which were common to both control strain datasets (Fig. 1A). DAVID functional annotation analysis showed no significantly enriched gene ontology (GO), Interpro protein domain or KEGG (Kyoto Encyclopaedia of Genes and Genomes) Pathway terms in the up-regulated genes. However, the down-regulated genes showed over-representation of a number of terms as shown in Table 1. The GO terms with the lowest P-values were ‘regulation of transcription, DNA dependent’, ‘regulation of RNA metabolic process’ and ‘ubiquitin-dependent protein catabolic process’ for Biological Process and terms relating to ‘transcription factor activity’ for Molecular Function. There were no GO terms for Cellular Component with a P-value less than 0.01, therefore any terms identified were discounted in this analysis. KEGG pathway analysis revealed a greater than 8-fold enrichment for ‘ubiquitin mediated proteolysis’ consistent with this dataset being enriched for genes related to the UPS. Interpro analysis showed over-representation of a number of protein domain classes including F-Box proteins, BTB proteins and SKp1-related (Skr) proteins which are all components of multimeric RING finger E3 ubiquitin ligase complexes. Additionally, DAVID Functional Annotation Clustering, which clusters genes based on functionally similar terms from the dataset, also identified an over-representation of F-Box related genes with a high enrichment score of 30.65.

Increasing the stringency by decreasing the FDR to 1% reduced the number of DEGs common to both controls to 31 upregulated and 182 down-regulated genes (Fig. 1A,B). Reanalysis of the down-regulated genes using DAVID Functional Annotation still revealed similarly enriched annotation terms for the UPS pathway in GO, KEGG Pathways and Interpro domains (Table 1). Notably the Biological Process GO term, ‘ubiquitin-dependent protein catabolic process’ had a lower P-value and higher fold enrichment whereas GO terms relating to the regulation of transcription were less significant in this smaller, more stringent dataset. DAVID Functional Annotation Clustering again showed enrichment of F-Box proteins (enrichment score = 29.35) and BTB/POZ domain proteins but the enrichment score for Transcription factors was only 2.55 (Table 2). Table 2 shows a list of down-regulated genes found in each of these three clusters from the more stringent 1% FDR dataset along with any known human homologs and the average fold decrease detected in the microarray.

### Regulatory motif analysis

The promoter sequences of DEGs from the 5% FDR dataset were analysed to identify any potential overrepresented regulatory motifs. The RSAT (Regulatory Sequence Analysis Tools) suite of programs were used to retrieve sequences 500 bp upstream of the transcription start site (TSS) of each gene and subsequently search for overrepresented oligonucleotides within these sequences. The top four most significantly overrepresented motifs identified in both the 5% FDR and 1% FDR datasets are shown in Table 3. The most significantly overrepresented motif was ATCGATA with over 29% of genes containing this motif. A second motif, ACGCTG, was also detected in over 20% of sequences. ATCGAT and TATCGA are accessory motifs which often co-occurred with ATCGATA and were found in ~40% of promoter sequences (Table 3 and Supplementary Table S2). Enrichment of both unique motifs was further confirmed using SCOPE motif finder. These motifs were submitted to TOMTOM for comparison against a database of known nematode motifs, however no significant matches were found. DAVID functional annotation clustering analysis showed enrichment for F-Box and BTB domains in the genes containing the motifs related to ATCGATA and also to a lesser extent in genes containing the ACGCTG motif (Table 3 and Supplementary Table S2).

### Functional validation of UPS pathway involvement

**Figure 2:**
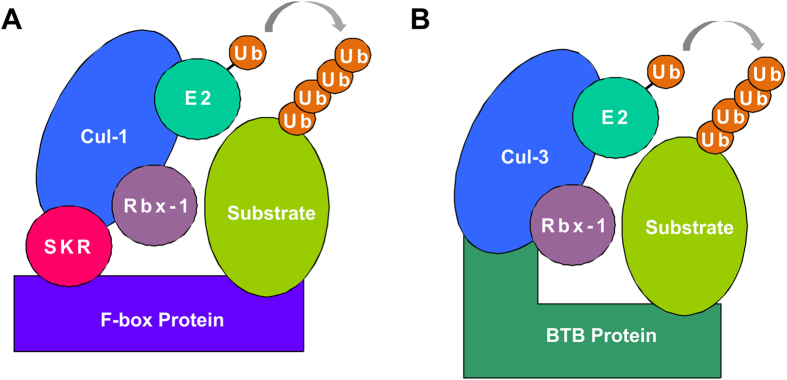
Schematic diagrams illustrating the composition of multimeric RING E3 Ubiquitin ligases. (**A**) SCF complex containing CUL1, (**B**) BTB complex containing CUL3.

Analysis of the microarray data revealed an enrichment for F-Box, SKR and BTB proteins which are all components of cullin-based multimeric RING finger E3 ubiquitin ligase complexes^32^. Cullins are a conserved family of E3 ligase components, of which there are 6 in *C. elegans* (CUL1 to 6). F-Box and SKR proteins are key components of SCF-type E3 ligase complexes which contain CUL1. SKR proteins bind to CUL1 and act as adaptor proteins recruiting an F-Box substrate-recognition subunit which positions target proteins to allow their ubiquitylation^32^ (Fig. 2A). BTB proteins, on the other hand, can act in a different type of E3 ligase complex which contains CUL3. In this complex the BTB protein acts as both the adaptor and the substrate-recognition subunit combined (Fig. 2B)^32^. 326 F-Box, 21 skr and over 100 btb genes have been described in *C. elegans*^32^ of which 44 F-Box, 7 skr and 16 btb transcripts were shown to be down-regulated in *dnj-14* mutant strains. This broad reduction in the transcription of E3-ligase related genes suggests that there may be an overall decrease in ubiquitin-proteasome pathway activity.

To investigate this, we studied the specific turnover of a GFP tagged ubiquitin-fusion degradation (UFD) substrate in N2 and *dnj-14 (tm3223)* worms. Follow up analyses were performed on *dnj-14 (tm3223)* worms only due to the confinement of the tm3223 mutation within the *dnj-14* gene compared to the ok237 deletion which also disrupts the neighbouring gene, *glit-1*.

PP563 worms stably express the UFD substrate, a non-cleavable ubiquitin fused N-terminally to GFP (UbV-GFP), under the *sur-5* promoter which is active in most tissues in *C. elegans*^33^. Under normal conditions UbV-GFP should become polyubiquitinated and targeted to the proteasome for degradation, however, if the UPS system is compromised this can lead to accumulation of UbV-GFP and can be measured by monitoring GFP fluorescence. UbV-GFP expression in *C. elegans* can be stabilized by mutating the ubiquitylation target residues K29 and K48 to arginine residues^33^. PP545 worms express ^K29,48R^UbV-GFP under the control of the sur-5 promoter. It has been previously demonstrated that inhibition of the proteasome through bortezamib treatment stabilizes UbV-GFP where as the drug has no effect on levels of the ^K29,48R^UbV-GFP variant which is constitutively accumulated^33^,^34^. We performed experiments to monitor the accumulation of UbV-GFP in PP563 and PP545 strains after overnight incubation with or without 10 μM bortezomib. There was no GFP detected in untreated PP563 worms but a signal was detectable after bortezomib treatment. In contrast, as expected, GFP was already accumulated in PP545 worms before treatment. Quantitation showed that the average GFP signal from PP545 worm lysates after bortezomib treatment was 94% +/− 0.005% (+/−SEM) compared to untreated PP545 worms (n = 3). This illustrates that non-ubiquitylated GFP would not be otherwise targeted to the proteasome when expressed under the *sur-5* promoter.

**Figure 3:**
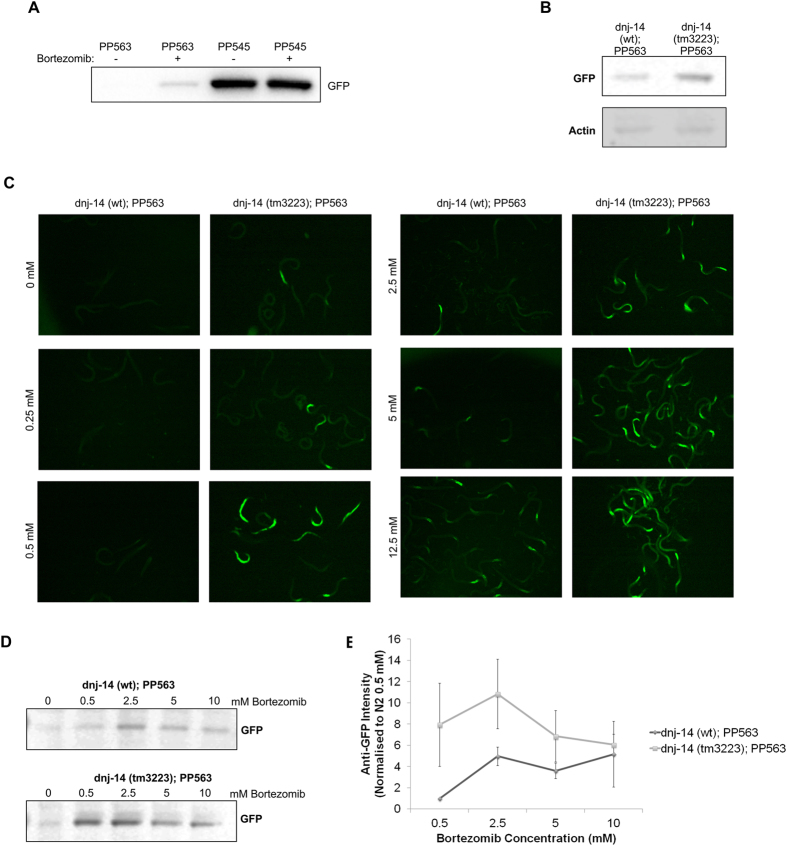
*dnj-14 (tm3223)* worms are delayed in ubiquitylated protein degradation (A) Representative image of Western blot detection of GFP-UbV accumulation in PP563 and PP545 strains after overnight incubation with or without 10 μM bortezomib. There was no GFP detected in untreated PP563 worms but a signal was detectable after bortezomib treatment. In contrast GFP was already accumulated in PP545 worms before treatment. Quantitation using ImageJ showed that the average GFP signal from PP545 worm lysates after bortezomib treatment was 94% +/− 0.005% (+/−SEM) compared to untreated PP545 worms (n = 3). (**B**) Representative image of Western blot detection of GFP-UbV accumulation in N2 and *dnj-14 (tm3223)* crossed with the pSur5::UbV-GFP expressing strain, PP563, generating the strains, *dnj-14 (wt)*; PP563 and *dnj-14 (tm3223)*; PP563. There was an average intensity increase of 354 +/−109% (+/−SEM) in *dnj-14 (tm3223)*; PP563 compared to *dnj-14 (wt)*; PP563 (n = 3). Worms were harvested and lysed at day 1 of adulthood. 50 worms were lysed in each SDS-PAGE sample and anti-beta-actin was used as a loading control. (**B**) GFP fluorescence was imaged in *dnj-14 (wt)*; PP563 and *dnj-14 (tm3223)*; PP563 worms after overnight incubation with increasing concentrations of bortezomib. (**C**) Western blot detection of GFP-UbV accumulation, using an anti-GFP antibody, after overnight treatment of *dnj-14 (wt)*; PP563 and *dnj-14 (tm3223)*; PP563 with increasing concentrations of bortezomib. (**D**) Quantitation of anti-GFP signal detected in (**C**). Band intensity for 0 mM was assumed to be background and was subtracted from the band intensity for drug treated samples for the corresponding strain. All data was then normalised against the signal for *dnj-14 (wt)*; PP563 at 0.5 mM. Error bars represent the standard error of the mean. Use of ANOVAR showed that data from the *dnj-14 (tm3223)* worms was statistically significantly different from the control worm data (n = 3, p < 0.02).

Next PP563 worms, were crossed with *dnj-14 (tm3223)* and lines selected based on the presence or absence of the tm3223 allele using PCR analysis. Immuno-blotting of lysates from these worms showed an increased accumulation of UbV-GFP in day 1 adult *dnj-14 (tm3223)*; PP563 worms compared to *dnj-14 (wt)*; PP563 as evidenced by detection with an anti-GFP antibody (average intensity increase of 354 +/− 109% (+/−SEM), Fig. 3A). UbV-GFP expressing worms were also challenged with the 26S proteasome inhibitor, bortezomib, overnight at concentrations ranging from 0 to 12.5 mM. Bortezomib treatment was used to enhance the observed proteasomal dysfunction and better report the extent to which UPS function is reduced in *dnj-14* mutant worms. GFP accumulation was detected either by imaging GFP fluorescence (Fig. 3B) or by immuno-blotting using an anti-GFP antibody (Fig. 3C,D). Again, higher GFP fluorescence was observed in *dnj-14 (tm3223)* worms compared to *dnj-14 (wt)* even in the absence of proteasome inhibitor (Fig. 3B). When challenged with proteasome inhibitor, *dnj-14 (tm3223)* worms exhibited accumulation of UbV-GFP at bortezomib concentrations of 0.5 mM whereas dnj-14 (wt) worms showed accumulation of UbV-GFP only at drug concentrations of 2.5 mM and above (Fig. 3B–D). *dnj-14 (tm3223)* worms also consistently showed a higher intensity of GFP in both imaging and immunoblotting experiments after bortezomib treatment (Fig. 3D, p < 0.02). This higher sensitivity to proteasome inhibition is indicative that ubiquitylated protein degradation is indeed compromised in *dnj-14 (tm3223)* worms. The downregulation of key components of E3 ubiquitin ligase complexes may have prevented proper ubiquitylation and efficient targeting of substrates for degradation by the proteasome.

## Discussion

A high representation of E3 ubiquitin ligase components, including skr genes, F-Box genes and btb domain containing genes, were observed to be down-regulated in *dnj-14* deficient animals compared to control nematodes in this study with these classes of genes representing 37% of the total down-regulated genes in the 1% FDR dataset. SKR, F-Box and BTB proteins are intrinsic to multimeric RING E3 ligases which contain CUL1 or CUL3 and bind a substrate protein bringing it into close proximity of an E2 ligase to allow ubiquitylation^35^. The broad down-regulation of these genes indicates an involvement for the UPS and protein homeostasis in CSP deficient worms. Due to the specific function of CSP in synaptic maintenance it would be interesting to investigate the contribution of neuronal-specific transcript alterations to the global changes observed in this microarray screen. Unfortunately, the small size of the nematode limits access to individual cell-types for molecular analysis and while some researchers have described methods to isolate specific cells in embryonic cultures^36^ and larvae^37^,^38^, this is not yet possible in adult populations which would be most applicable to our aging and neurodegeneration model.

Analysis of the promoter sequences for DEGs common to both control sets revealed two potential regulatory motifs, ATCGATA and ACGCTG. Studies identifying DNA binding motifs for transcription factors in *C. elegans* is currently limited and comparison to a database of known binding motifs did not identify any potential *C. elegans* regulatory factors. When literature searches were performed, however, the mammalian transcription factor, Cux1, was found to bind to the ATCGAT motif. Cux1 is a CUT homeodomain protein which is known to have the consensus sequence ATCGAT^39^. *ceh-44* is an orthologue of Cux1 in *C. elegans* and was found to be down-regulated in *dnj-14* mutants compared to both of our control microarray datasets. In *Drosophila* the Cux1 homolog, Cut, functions as a determinant of cell-type specification of a number of tissues including in the peripheral nervous system and is a target of the Notch signalling pathway^40^.

Our research also revealed that *dnj-14* mutant worms expressing a GFP tagged UFD substrate showed higher sensitivity when challenged with the proteasomal inhibitor, bortezomib, as evidenced by the accumulation of UbV-GFP at lower drug concentrations than in control worms. This effect was apparent in day 1 worms indicating that alterations in UPS activity, in accordance with the broad down-regulation of UPS-related genes in older worms, were evident even in young adult worms. To try to address whether there is also an increased sensitivity of the UPS system specifically in the nervous system of *dnj-14* mutant worms we have also attempted to express UbV-GFP under the control of the *rab-3*, pan-neuronal promoter. Unfortunately, due to high expression levels of the reporter construct, we observed constitutive accumulation of the UbV-GFP probe even in wildtype nematodes, thus hindering this line of inquiry.

The observed changes to the UPS in our study appear at odds with previous observations from CSP KO mice where the expression of proteins involved in proteasomal degradation was increased and a broad enhancement of proteasomal activity was observed^21^. This observation was based on immunoblotting for a select few proteasomal proteins and fluorometry-based activity assays of the proteasomal active sites but did not examine components of multimeric E3 ubiquitin ligases. It is possible, therefore, that the increased breadth and sensitivity offered by microarray analysis has highlighted subtle changes in the *C. elegans dnj-14* deficient models that were not identified in CSP KO mice.

In CSP KO mice, an increase in UPS-related protein expression was observed, which was coupled with a ~40% increase in SNAP-25 ubiquitylation and enhanced proteasomal activity^21^. Misfolded neurotoxic proteins are often ubiquitylated and targeted for degradation in age-related neurological disorders and it is thought that this might be a protective mechanism by which brain cells try to cope with neurodegeneration. Sharma *et al.,* however, observed a therapeutic effect of proteasomal inhibitors, lactacystin and epoxomicin, in CSP-depleted mice due to a ~2-fold increase in levels of SNAP-25 and SNARE complex assembly^21^. Conformational quality control of SNAP-25 is crucial for the maintenance of high frequency synaptic activity. This quality control process exists in equilibrium between chaperone mediated refolding by CSP which promotes SNARE complex assembly, and UPS-dependent degradation which reduces SNAP-25 levels and hence decreases synaptic activity. In CSP deficient mice, proteasomal inhibition can shift the balance away from SNAP-25 degradation and help maintain more functional SNARE complexes and hence synaptic activity^21^. We tested the effect of proteasome inhibition in *dnj-14 (tm3223)* worms by monitoring the lifespan of nematodes grown in the presence of either 0.5 μM or 2.5 μM bortezomib, however, we did not observe any significant difference compared to vehicle treated controls (data not shown). In *C. elegans* it appears that a protective mechanism may have evolved naturally through the downregulation of UPS-pathway genes without the need for additional proteasome inhibitor treatment. Selective pressures acting on the *dnj-14* mutant worms over many generations may have allowed a compensatory reduction in UPS activity which would not be possible in first generation CSP^−/−^ KO mouse models. We propose that a reduction in the expression of RING finger E3 ubiquitin ligase components reduces the ubiquitylation and degradation of essential synaptic proteins such as RIC-4, the *C. elegans* homolog of SNAP-25. Unfortunately, this theory could not be directly tested as both a polyclonal SNAP-25 antibody and a custom made polyclonal RIC-4 antibody, failed to give a specific signal when *C. elegans* lysates were immunoblotted.

The action of E3 ligases in different organisms has been previously shown to affect the stability and localisation of various aging-related genes including almost all components of the insulin/insulin-like growth factor-1 (IGF-1)-signalling (IIS) pathway^41^. Additionally, A number of studies have identified connections between the UPS pathway and age-related processes in *C. elegans*. Ghazi *et al.* previously performed an RNAi screen of *C. elegans* proteasomal subunits and SCF complex components to identify factors which affect lifespan^42^. In this screen they found that a CUL1 E3 ligase SCF complex is required for lifespan extension of *C. elegans*’ IIS pathway mutants. They speculate that this CUL1 complex could ubiquitylate and degrade one or more proteins that actively regulate the rate of aging^42^. In another study Kuhlbrodt *et al.* found that loss of a deubiquitinating enzyme, ATX-3, and the ubiquitin-selective chaperone, CDC-48, leads to lifespan extension by stabilizing components of the IIS pathways thus activating transcription of DAF-16 targets^43^.

A study examining the gene profile of *C. elegans* at mid-life transition (day 5 adult worms) identified a group of genes and compared these to those which had been found in other age-related studies^44^. Of the total DEGs identified in the present study 19 (6.6%) were present within the combined analysis reported by Eckley *et al.* These authors suggest that targeted proteolysis is essential during the onset of senescence in post-developmental animals and 42% of the differentially expressed UPS related genes at midlife transition were common to those differentially expressed in *dnj-14* mutants. While there is, therefore, some overlap with known ageing-related genes, many of the differentially regulated UPS-related genes identified in *dnj-14* mutants are unique to our study. This indicates that the changes were not simply a consequence of premature aging in *dnj-14* mutants and alternative non-ageing related processes might be activated in these worms.

This study has revealed a genetic interaction between *dnj-14* and UPS system regulation resulting in down-regulation of multiple E3 ligase components and decreased ubiquitylated protein degradation. The UPS has received a lot of attention in relation to the study of neurodegenerative diseases however there has been a lack of consensus about whether UPS activity is increased or decreased in these disorders^2^. Likewise, reports are varied about whether direct proteasomal inhibition is detrimental or beneficial depending on the disease or system being studied^2^. For example, inhibition of proteasome activity has been shown to be beneficial in protection of β-amyloid neurotoxicity^45^, as well as in the CSP-deficient mouse model. Treatment with proteasomal inhibitors can, however, itself result in neurodegeneration^46^. Instead, our results hint that therapeutics specifically targeting proteins and complexes, such as E3 ubiquitin ligases, which act upstream of the 26S proteasome, may be beneficial as potential pharmacological targets for the treatment of neurodegenerative diseases including ANCL. Indeed, a number of ubiquitin ligases have been identified as promising drug targets^47^,^48^,^49^ and progress has been made toward developing specific inhibitors of SCF complexes in particular^50^,^51^,^52^,^53^,^54^,^55^,^56^,^57^.

## Methods

### Strains

*C. elegans* strains RM2754 *dnj-14 (ok237)* and CZ1200 juIs76 [unc-25p::GFP + *lin-15*(+)] II were obtained from the *Caenorhaditis* Genetics Center (CGC; University of Minnesota, Twin Cities, MN, USA). The TM3223 *dnj-14 (tm3223)* strain was obtained from the National Bioresource Project for the Experimental Animal “Nematode *C. elegans*” based in the lab of Dr Shohei Mitani (Tokyo Women’s Medical University, Tokyo, Japan). PP563 *unc-119*(ed4)III; hhIs64 [unc-119(+); sur-5::UbV-GFP]III and PP545 unc-119(ed4)III; hhIs53 [unc-119(+); sur-5::K29/48R-UbV-GFP] were a kind gift from the lab of Professor Hoppe (University of Cologne, Germany).

### Strain Construction and Verification

TM3223 transgenic strains expressing sur-5::UbV-GFP were generated by mating the TM3223 *dnj-14(tm3223)* with PP563 unc-119(ed4)III; hhIs64 [*unc-119*(+); sur-5::UbV-GFP]III. Worms expressing GFP were selected and PCR was used to verify the presence of the tm3323 allele using primers as described previously^25^.

### Nematode culture

*C. elegans* were grown at 20 °C under standard conditions on nematode growth media (NGM; 2% (w/v) agar, 0.3% (w/v) NaCl, 0.25% (w/v) peptone, 1 mM CaCl_2_, 5 μg ml^−1^ cholesterol, 25 mM KH_2_PO_4_, 1 mM MgSO_4_) agar plates. *Escherischia coli* OP50 was used as a food source. All experiments were performed at 20 °C.

### Microarray Analysis

N2, CZ1200, *dnj-14 (ok237)* and *dnj-14 (tm3223)* strains were first age synchronised by bleaching. Worms were then grown for 2 days after L4 stage (day 2 of adulthood) and 50 hermaphrodites of each strain were transferred to new plates and allowed to lay eggs for 6 hours before removal. After hatching worms were grown until L4 stage then 400 hermaphrodites from each strain were picked onto fresh plates. Worms were transferred onto fresh plates on alternate days until day 6 of adulthood when worms were harvested by washing plates with DNase/RNase-free distilled water (Invitrogen). Total RNA was extracted using TRIzol reagent (Invitrogen) and purified using Qiagen RNAeasy Mini Kit (Qiagen). Three biological replicates were prepared for each of the four strains used and hybridised to Affymetrix *C. elegans* GeneChip arrays according to the manufacturer’s instructions.

### Data generation and diagnostic analysis

Initial diagnostic and differential expression analysis of the microarray data was performed using the statistical analysis tools R, Affy (version 2.15.2) and limma (version 3.14.4). Background correction and normalisation of raw Affymetrix array data were conducted using the Robust Multi-array Average function built into the Affy package^58^. The significance test of the estimated logFC (log_2_ Fold Change) for each contrast was performed by the empirical Bayes function packed in limma and *p*-values were adjusted using the Benjamini and Hochberg FDR control approach to deal with the effect of multiple tests^59^.

### Gene Ontology Analysis

Data were analysed using the Database for Annotation, Visualization and Integrated Discovery (DAVID) V6.7 from the National Institutes of Health^60^,^61^. A gene list for analysis was generated by submitting the affymetrix *C. elegans* probe IDs of commonly regulated DEGs to DAVID for conversion to Ensembl Gene IDs and any duplicates were removed. For GO Term analysis, the “Biological Process”, “Cellular Component” and “Molecular Function” categories were studied using the GO FAT default settings. Protein domain and pathway enrichment were investigated using DAVID by querying the Interpro and KEGG databases, respectively. Functional annotation clustering was performed with the default criteria at medium classification stringency. Human orthologues of the corresponding worm genes were derived from either wormbase^62^ or the OrthoList online tool^63^.

### Motif analysis

The Regulatory Sequence Analysis Tools suite (RSAT)^64^ was first used to retrieve sequences 500 bp upstream of the TSS for all genes in the 5% FDR dataset. This was subsequently submitted to the RSAT oligo analysis tool^65^ to identify overrepresented motifs in the sequences. To confirm enrichment of these motifs, gene names were submitted to SCOPE motif finder^66^ specifying sequences 400 bp upstream of the TSS. In each case sequences were examined for oligonucleotides of 6–8 bases in length.

### Bortezomib experiments

To test the effect of bortezomib treatment on PP563 and PP545 strains, 50 worms of either strain were picked into tubes of 100 μl S-Basal solution (100 mM NaCl, 6 mM K_2_HPO_4_, 44 mM KH_2_PO_4_, 5 μg/ml cholesterol) containing OP50 as a food source, with or without 10 μM bortezomib. Worms were incubated with gentle shaking overnight at 20 °C. Worms were then washed in S-basal solution and the worms were allowed to settle to the bottom of the tube. Excess supernatant was then removed and worms were analysed by SDS-polyacrylamide gel electrophoresis (SDS-PAGE). Bortezomib dose response experiments were conducted in the same way using either *dnj-14 (wt)* or *dnj-14 (tm3223)* stably expressing pSur5::Ubv-GFP and increasing concentrations of bortezomib as indicated in the figure legends. Worms were then either imaged or analysed by SDS-polyacrylamide gel electrophoresis (SDS-PAGE). As treatment with increasing levels of proteasome inhibitor will affect the levels of many proteins within the cell, equal loading was assessed and confirmed by Ponceau S staining of nitrocellulose membranes prior to western blotting to visualize protein levels in whole lanes rather than blotting for a single housekeeping protein (see Supplementary Figure S2).

### Imaging

Worms were picked into a droplet of Dents buffer (140 mM NaCl, 6 mM KCl, 1 mM CaCl2, 1 mM MgCl2, 5 mM HEPES, pH 7.4 with bovine serum albumin at 0.1 mg/ml) before imaging using a Nikon Eclipse Ti S fluorescence microscope with a × 20 objective and processed using the software NIS Elements.

### Protein electrophoresis and western blot analysis

Worm lysates were prepared in SDS loading buffer (4% SDS, 20% glycerol, 10% 2-mercaptoethanol, 0.004% bromophenol blue and 0.125 M Tris HCl, pH 6.8), vortexed for 1 minute before freezing at −80 °C and boiled at 95 °C for 20 minutes. Samples were separated by SDS-PAGE on NuPAGE® Novex® 4–12% bis-tris gels using NuPAGE® MOPS SDS running buffer (Invitrogen). The fractionated proteins were then transferred to nitrocellulose membranes, which were then blocked in 0.5% fish skin gelatin (sigma) in PBS for 1 hour at room temperature. The membranes were subsequently incubated with mouse anti-GFP (roche) for 1 hour at room temperature. Membranes were subsequently incubated with mouse anti-beta-actin (sigma) for 1 hour at room temperature as a loading control. IRDye conjugated secondary antibody, anti-mouse 680RD (LI-COR), was incubated with membranes for 1 hour at room temperature before imaging with an Odyssey Sa infrared imaging system (LI-COR). The relative abundance of GFP levels was quantified using either Image Studio Lite (LI-COR) or ImageJ.

## Additional Information

**How to cite this article**: McCue, H. V. *et al.* Expression profile of a *Caenorhabditis elegans* model of adult neuronal ceroid lipofuscinosis reveals down regulation of ubiquitin E3 ligase components. *Sci. Rep.* **5**, 14392; doi: 10.1038/srep14392 (2015).

## Supplementary Material

Supplementary Figures and Information

Supplementary table 1

Supplementary table 2

## Figures and Tables

**Table 1:** David Functional Annotation for the down-regulated genes in 5% and 1% FDR datasets.

	Annotation Term	5% FDR		1% FDR	
		P-Value	Fold Enrichment	P-Value	Fold Enrichment
Biological Process	GO:0006355~regulation of transcription, DNA-dependent	1.33E-04	2.80	0.003985128	2.64
	GO:0051252~regulation of RNA metabolic process	1.40E-04	2.78	0.004123515	2.63
	GO:0006511~ubiquitin-dependent protein catabolic process	1.49E-04	8.51	2.45E-04	10.32
	GO:0045449~regulation of transcription	4.50E-04	2.43	0.016440945	2.17
	GO:0044265~cellular macromolecule catabolic process	0.0014285	4.67	0.006409969	4.96
	GO:0019941~modification-dependent protein catabolic process	0.002640061	4.94	0.002855343	6.00
	GO:0043632~modification-dependent macromolecule catabolic process	0.002640061	4.94	0.002855343	6.00
	GO:0042464~dosage compensation, by hypoactivation of X chromosome	0.003032918	34.84	—	—
	GO:0051603~proteolysis involved in cellular protein catabolic process	0.003323413	4.72	0.003485241	5.72
	GO:0044257~cellular protein catabolic process	0.003430888	4.69	0.003582778	5.69
	GO:0007549~dosage compensation	0.003768035	31.35	—	—
	GO:0009057~macromolecule catabolic process	0.003815176	3.93	0.013037275	4.17
	GO:0030163~protein catabolic process	0.005373442	4.28	0.005292688	5.19
	GO:0010171~body morphogenesis	0.007835542	2.33	—	—
	GO:0007530~sex determination	0.049688021	8.25	—	—
	GO:0006508~proteolysis	0.054970961	2.01	0.020161301	2.56
Molecular Function	GO:0003700~transcription factor activity	6.23E-05	3.08	1.19E-03	3.04
	GO:0030528~transcription regulator activity	7.45E-05	2.77	6.70E-03	2.44
	GO:0003677~DNA binding	7.43E-04	2.23	7.13E-03	2.18
KEGG Pathway	cel04120:Ubiquitin mediated proteolysis	5.46E-05	8.32	2.42E-05	11.89
Protein Domain (Interpro)	IPR012885:F-Box associated type 2	3.15E-42	21.09	1.50E-41	24.24
	IPR001810:Cyclin-like F-Box	7.41E-30	9.37	3.79E-28	10.30
	IPR011333:BTB/POZ fold	2.87E-10	8.91	1.88E-09	9.66
	IPR001699:Transcription factor, T-box	2.08E-09	33.51	3.46E-05	25.97
	IPR016897:E3 ubiquitin ligase, SCF complex, Skp subunit	3.13E-08	34.20	4.47E-07	36.35
	IPR018186:Transcription factor, T-box, conserved site	6.41E-08	30.78	7.48E-04	21.81
	IPR016072:SKP1 component, dimerisation	6.41E-08	30.78	7.98E-07	32.72
	IPR001232:SKP1 component	1.62E-07	26.77	1.69E-06	28.45
	IPR016073:SKP1 component, POZ	2.79E-07	24.63	2.64E-06	26.18
	IPR000571:Zinc finger, CCCH-type	4.26E-05	15.08	1.50E-05	18.70
	IPR000210:BTB/POZ-like	2.50E-04	5.42	3.71E-04	5.98
	IPR013069:BTB/POZ	0.005488655	5.28	2.17E-03	6.54

**Table 2:** DAVID Functional Annotation Clustering gene list for down-regulated genes in the 1% FDR dataset.

Cluster	Ensembl ID	Gene Name	Human Orthologue	Average Fold Change
F-BOX proteins (Enrichment Score = 29.35)	F58E1.5	*fbxb-17*	—	2.58
	M116.4	*sdz-25*	—	2.16
	K05F6.7	*fbxb-54*	—	2.08
	F53C3.2	*fbxb-103*	—	2.00
	Y51H7BR.2	*fbxb-43*	—	1.97
	R17.1	*fbxb-85*	—	1.97
	F08D12.10	*sdz-9*	—	1.97
	Y43B11AL.1	*Y43B11AL.1*	—	1.91
	T26E3.5	*T26E3.5*	—	1.87
	ZC204.7	*fbxb-15*	—	1.87
	R08C7.9	*fbxb-74*	—	1.81
	F45C12.5	*fbxb-11*	—	1.80
	F58E1.14	*fbxb-47*	—	1.80
	W04A8.5	*W04A8.5*	—	1.78
	F58E1.8	*fbxb-18*	—	1.78
	M01D1.8	*fbxb-41*	—	1.78
	T08E11.6	*fbxb-10*	—	1.74
	F55C9.13	*fbxb-63*	—	1.73
	ZC204.9	*fbxb-20*	—	1.70
	F56G4.3	*pes-2.1*	—	1.68
	F12E12.10	*fbxb-90*	—	1.68
	M151.5	*fbxb-31*	—	1.66
	W04A8.3	*W04A8.3*	—	1.65
	C52E2.1	*fbxb-95*	—	1.65
	C39B5.2	*C39B5.2*	—	1.64
	Y46G5A.8	*Y46G5A.8*	—	1.63
	H24O09.2	*fbxb-72*	—	1.60
	F36H5.8	*F36H5.8*	—	1.60
	T26E3.8	*T26E3.8*	—	1.59
	F08D12.9	*sdz-10*	—	1.53
	F55C9.8	*fbxb-62*	—	1.51
	F45D11.13	*fbxb-30*	—	1.50
	F49B2.2	*fbxb-67*	—	1.48
	T16A1.8	*fbxb-37*	—	1.48
	Y113G7B.8	*fbxb-59*	—	1.44
	T17A3.4	*fbxb-82*	—	1.43
	C08F1.3	*fbxb-13*	—	1.41
	C36C9.3	*fbxa-170*	—	1.40
BTB/POZ domain proteins (Enrichment Score = 4.22)	M01D1.3	*btb-11*	—	2.07
	R52.1	*sdz-28*	Isoform 2 of Kelch-like protein 28	2.04
	K02E7.9	*btb-10*	—	1.87
	Y105C5B.13	*skr-10*	Isoform 1 of S-phase kinase-associated protein 1	1.87
	C52D10.8	*skr-13*	Uncharactersised protein	1.85
	B0281.5	*B0281.5*	potassium channel tetramerization domain containing 10	1.85
	F54D10.1	*skr-15*	Uncharactersised protein	1.75
	C52D10.7	*skr-9*	Isoform 1 of S-phase kinase-associated protein 1	1.74
	Y47D7A.8	*skr-14*	Uncharactersised protein	1.69
	F45C12.6	*btb-8*	—	1.64
	C52D10.9	*skr-8*	Isoform 1 of S-phase kinase-associated protein 1	1.61
	C40A11.2	*C40A11.2*	potassium channel tetramerization domain containing 10	1.54
	ZC204.3	*btb-12*	—	1.50
	F45C12.7	*btb-6*	—	1.50
Transcription Factors (Enrichment Score = 2.55)	Y47D3A.12	*tbx-37*	T-box transcription factor TBX21	2.26
	ZK662.4	*lin-15B*	Isoform 1 of Uncharacterized protein C6orf132	2.23
	C24H11.3	*tbx-38*	TBX6	2.00
	Y46E12A.4	*Y46E12A.4*	Eomesodermin homolog	1.88
	F17A2.5	*ceh-40*	pre-B-cell leukemia homeobox 1	1.68
	F40H6.4	*tbx-11*	T-box transcription factor TBX21	1.67
	C29F7.4	*fkh-3*	Uncharactersised protein	1.67
	C32F10.6	*nhr-2*	highly similar to Retinoic acid receptor gamma-2	1.63
	C50F4.6	*his-41*	Histone H2B type 1-L	1.57
	F19F10.5	*ets-7*	Isoform 2 of ETS domain-containing protein Elk-4	1.56
	C30A5.2	*rfs-1*	Isoform 1 of DNA repair protein RAD51 homolog 4	1.41
	F19B10.9	*sea-1*	Isoform 1 of T-box transcription factor TBX22	1.37
	ZC376.4	*ceh-74*	NOBOX oogenesis homeobox	1.29
	ZC204.2	*duxl-1*	double homeobox protein 4-like protein 4	1.27

The enrichment score for each cluster is given in the first column and the average fold decrease for the two *dnj-14* mutants compared to the two control strains is given in the last column.

**Table 3:** Regulatory motif analysis.

Motif	ID	Exp Occ (%)	5% FDR			1% FDR			Enrichment Score	
			Occ (%)	P-Value	Score	Occ (%)	P-Value	Score	F-Box	BTB
atcgata	atcgata|tatcgat	8	29	1.70E-26	21.84	33	1.40E-21	16.95	14.24	2.98
atcgat	atcgat|atcgat	15	39	3.10E-23	19.19	44	6.50E-21	16.87	19.4	4.53
tatcga	tatcga|tcgata	20	40	3.80E-17	13.11	43	1.20E-17	13.62	22.47	3.39
acgctg	acgctg|cagcgt	6	20	1.10E-14	10.65	23	4.90E-15	11	3.47	—

The four most significantly overrepresented motifs in both the 5% and 1% FDR datasets (up- and down-regulated genes combined) are shown in this table. Lists of genes containing each of these motifs were analysed by DAVID functional clustering analysis and an enrichment score for F-Box and BTB proteins was generated. Exp Occ = expected occurrence, Occ = actual occurrence. The score was generated by RSAT indicating the degree of overrepresentation in the dataset.
