# Supplementary material for: Expression profile of a Caenorhabditis elegans model of adult neuronal ceroid lipofuscinosis reveals down regulation of ubiquitin E3 ligase components
Source: Sci Rep. 2015 Sep 23;5:14392. doi: 10.1038/srep14392 (PMC4585785; doi:10.1038/srep14392)
Supplement: Supplementary Figures and Information [file srep14392-s1.pdf]

Expression profile of a *Caenorhabditis elegans* model of adult neuronal ceroid lipofuscinosis reveals down regulation of ubiquitin E3 ligase components.

Hannah V. McCue[1*], Xi Chen[#], Jeff W. Barclay[1], Alan Morgan[1] and Robert D. Burgoyne[1]

SUPPLEMENTARY FIGURES AND TABLE LEGENDS

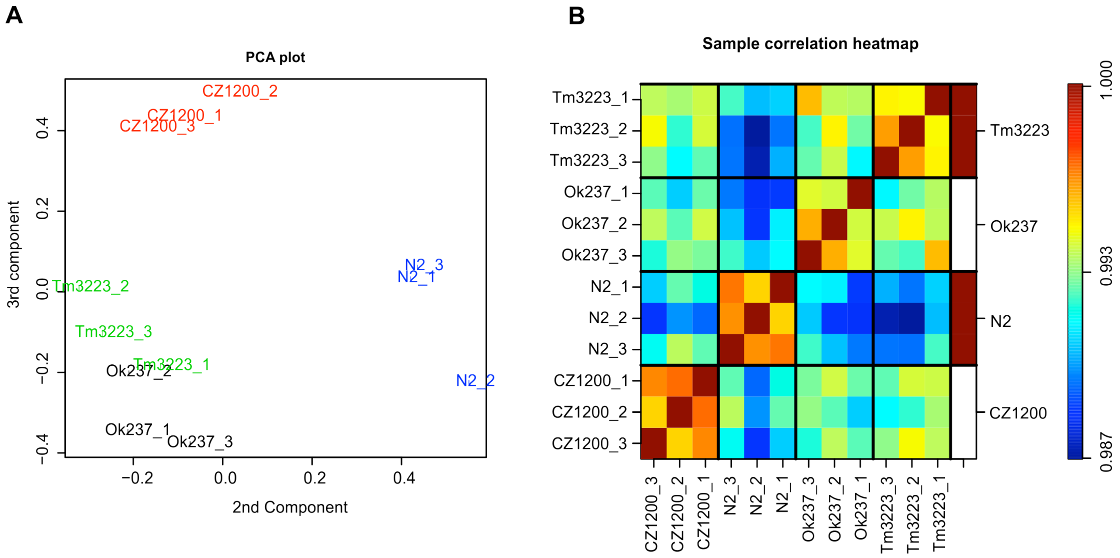

Supplementary Figure S1

Whole-genome expression profiling of *dnj-14(ok237), dnj-14(tm3223)*, CZ1200 and N2 strains. A) Principal component analysis (PCA) plot of transcription profiles of all four strains. The PCA plot shows the distance between profiles and denotes the similarities or dissimilarities between each strain. All three biological replicates in each condition show a high reproducibility. (B) Pearson correlation coefficient-based heat-map of expression similarity and divergences among the four strains based on genes that showed significant correlations. All three biological replicates are shown independently for each condition. Samples are clustered along both axes based on their correlation value and the diagonal of the heat-map indicates the amount of the

Pearson correlation between corresponding sample types. High covariance is indicated in dark red, low covariance is indicated in dark blue. Yellow and green regions indicate weak correlations/uncorrelated assets (see colour bar).

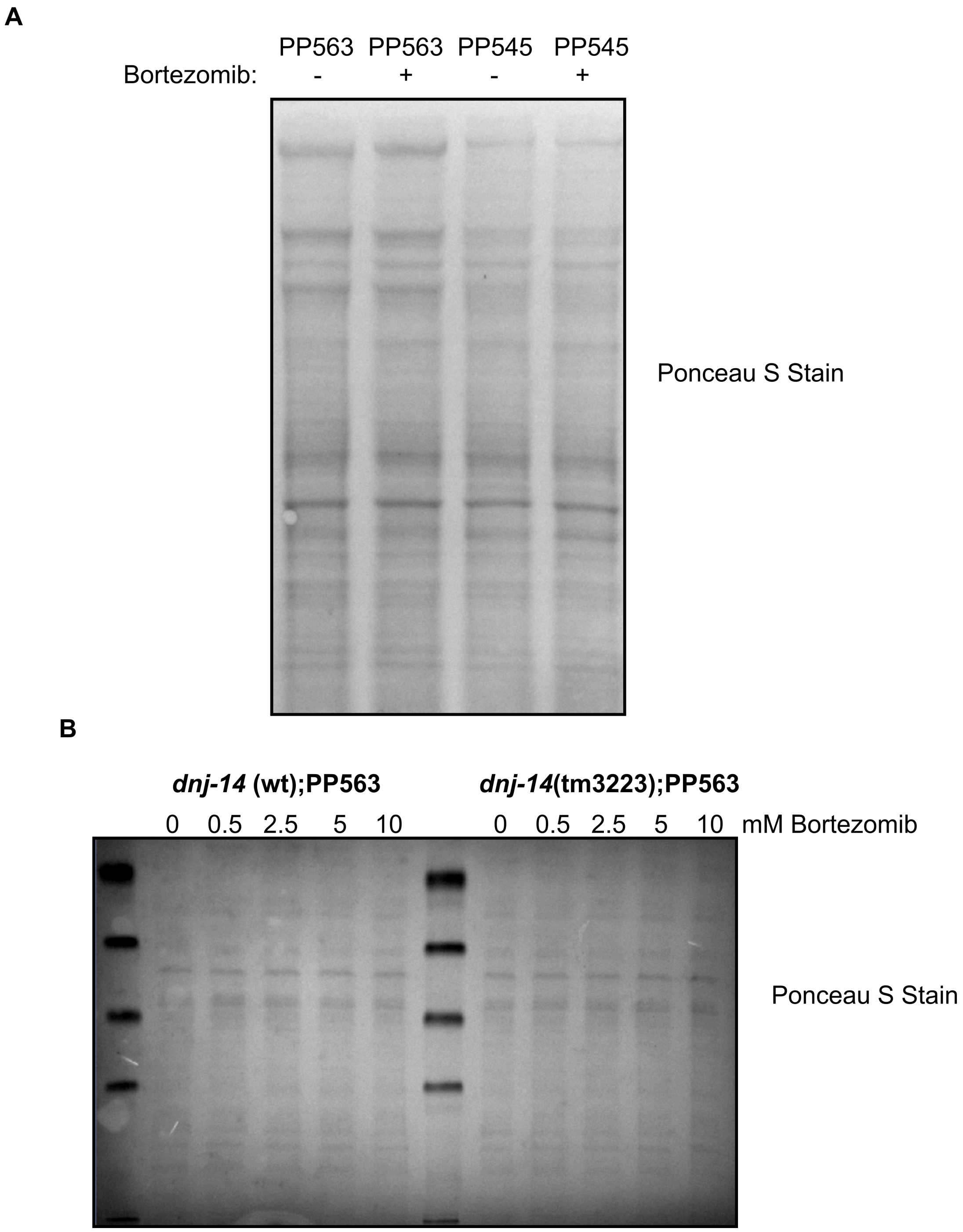

Supplementary Figure S2

Representative loading controls for figures 3A and 3D. A) Ponceau S stained nitrocellulose membrane showing equal loading of PP563 and PP545 samples with

and without bortezomib treatment as in figure 3A. B) Ponceau S stained nitrocellulose membrane showing equal loading of samples from a bortezomib dose response experiment as seen in figure 3D.

Supplementary Table S1.xls

Differentially expressed genes. Gene list of differentially expressed genes which were up or down regulated in both *dnj-14 (tm3223)* and *dnj-14 (ok237)* compared to both controls, N2 and CZ1200.

Supplementary Table S2.xls

Regulatory motif analysis. List of genes containing the top four most significantly overrepresented motifs within their promoter regions (500bp upstream of the TSS). Y = motif is found in this promoter sequence. N = motif is not found in this promoter sequence.
